# Identification of Key Genes Mutations Associated With the Radiosensitivity by Whole Exome Sequencing in Pancreatic Cancer

**DOI:** 10.3389/fonc.2021.697308

**Published:** 2021-08-09

**Authors:** Bin Hu, Xiumei Ma, Renhua Huang, Zheng Wu, Jun Lu, Yuntao Guo, Jianmin Tang, Chunhui Ma, Jun Ma, Lei Zhang, Yongrui Bai

**Affiliations:** ^1^Department of Radiation Oncology, Renji Hospital, School of Medicine, Shanghai Jiao Tong University, Shanghai, China; ^2^Department of Research, Medical Laboratory of Nantong Zhongke, Nantong, China; ^3^Department of Bioinformatics, Medical Laboratory of Nantong Zhongke, Nantong, China; ^4^Department of Orthopedic Surgery, Shanghai General Hospital, Shanghai Jiao Tong University, Shanghai, China; ^5^Eye Institute, Eye & ENT Hospital, Shanghai Medical College, Fudan University, Shanghai, China

**Keywords:** pancreatic cancer, radiotherapy, radioresistance, biomarker, Smad

## Abstract

**Background:**

Pancreatic cancer (PC) is one of the most lethal human cancers, and radiation therapy (RT) is an important treating option. Many patients diagnosed with PC do not achieve objective responses because of the existence of intrinsic and acquired radioresistance. Therefore, biomarkers, which predict radiotherapy benefit in PC, are eagerly needed to be identified.

**Methods:**

Whole-exome sequencing of six pancreatic ductal adenocarcinoma patients (PDAC) (three with a good response and three with a poor response) who had received radical surgery and then radiotherapy has been performed as standard of care treatment. Somatic and germline variants and the mutational signatures were analyzed with bioinformatics tools and public databases. Functional enrichment and pathway-based protein-protein interaction analyses were utilized to address the possibly mechanism in radioresistance. MTT, LDH, and colony formation assay were applied to evaluate cell growth and colony formation ability.

**Results:**

In the present study, somatic mutations located in 441 genes were detected to be radiosensitivity-related loci. Seventeen genes, including the Smad protein family members (SMAD3 and SMAD4), were identified to influence the radiosensitivity in PDAC. The SMAD3 and SMAD4 genes mutate differently between radiosensitive and radioresistant PDAC patients. Mutation of SMAD3 potentiates the effects of ionizing radiation (IR) on cell growth and colony formation in PDAC cells, whereas mutation of SMAD4 had the opposite effects. SMAD3 and SMAD4 regulate the radiosensitivity of PDAC, at least in part, by P21 and FOXO3a, respectively.

**Conclusions:**

These results indicate that mutations of SMAD3 and SMAD4 likely cause the difference of response to radiotherapy in PDAC, which might be considered as the biomarkers and potential targets for the radiotherapy of pancreatic cancer.

## Introduction

Pancreatic cancer (PC) is the fourth leading cause of tumor-related deaths presently, which is predicted to be the second most fatal cancer in 2030 (1, 2). In recent years, some treatment methods have been developed, and the comprehension for cancer biology is deepened. The survival rates of patients with cancer are improved by many new treatment modalities, such as targeted therapy, immunotherapy, and advanced radiological techniques. However, the prognosis of pancreatic cancer remains poor. Its overall survival rate is only around 10% for 1 year and less than 5% for 5 years (3). Pancreatic cancer patients that can be treated with surgical management only accounts for less than 20%. Therefore, the main treatment options for PC are still chemotherapy (CT) and radiation therapy (RT) (4). So far, 40% of cured patients has received radiotherapy (5). However, many PC patients failed to obtain objective therapeutic effect because of inherent or acquired radiation resistance.

Previous reports have proved that the efficiency of radiation therapy could be affected by a series of alterations concerning the tumor itself and the surrounding microenvironment, such as inhibition of apoptosis, increased DNA repair, inflammation, and hypoxia (6). Furthermore, lots of pathways, such as PI3K/AKT (7), Notch (8), Foxo (9), ATM/ATR (10), MEK/ERK (11), TGFβ (12, 13), Wnt (14), and genes, such as SMAD4 (15), MUC1 (16), RAD54 (17), have been identified to regulate the endogenous signals and contribute to resistance to radiation therapy (RI) in some cancers. Nevertheless, the molecular mechanisms of radioresistance in PC remain to be unravelled and in urgent need of further exploration.

To identify the critical mutations of affecting radiotherapy benefit in PC, whole exome sequencing was utilized in six pancreatic ductal adenocarcinoma (PDAC) patients (three with a good response and three with a poor response) who had received radical surgery. We aimed at finding novel therapeutic response predictive biomarkers and providing the basis for developing novel radiosensitizers.

## Materials And Methods

### Patients and Tissue Samples

Tumor and blood samples were collected from six Chinese patients diagnosed with PC. Briefly, radical surgery (total or partial gastrectomy) was performed on all six patients and the tumor tissues and blood samples were collected for analyzes. After resection, all patients received postoperative adjuvant chemotherapy (gemcitabine+tegafur, gimeracil, and oteracil porassium capsules), and then radiotherapy was further utilized for therapy. After radiotherapy, the patients continued to receive chemotherapy (tegafur, gimeracil, and oteracil porassium capsules). The original clinical and survival data of patients are shown in **Table 1**. We divided the patients into two group based on their responses to radiotherapy. Patients with no response to radiotherapy were considered as the radioresistant group (group N), and patients with complete response were considered as the sensitive group (group S). The studies were approved by the ethical committee of Ren Ji Hospital, Shanghai Jiao Tong University School of Medicine, and all patients signed informed consent in advance of their recruitment.

**Table 1:** Clinicopathological Characteristics of Six Primary Pancreatic Tumor Samples.

Patient_ID	Age	Gender	TNM	Specimen sites	Histology	Dose (Gy/Fx)	Stage	Status	OS	PFS
N1	71	F	pT4N0M0	Head	PDAC	2	III	die	13.53	8.57
N2	63	F	pT2N0M0	Body	PDAC	2	IB	die	19.43	8.33
N3	65	M	pT2N1M0	Head	PDAC	2	IIB	die	28.70	10.93
S1	77	M	pT3N0M0	Body	PDAC	1.8	IIA	die	49.17	30.60
S2	57	M	pT4N0M0	Body	PDAC	1.8	III	die	24.90	23.73
S3	63	M	pT3N1M0	Body	PDAC	2	IIB	alive	73.53	72.87

### DNA Extraction and Whole-Exome Sequencing

GenRead™ DNA FFPE Kit (Qiagen; Germany) was used to extract genomic DNA from formalin-fixed paraffin-embedded tissues and paired normal blood DNA. Then the genomic DNA was fragmented and hybridized following instructions on manufacturer’s protocol. These two methods were combined to verify the quality of isolated genomic DNA: 1) measuring DNA concentration using Qubit^®^ DNA Assay Kit in Qubit^®^ 2.0 Flurometer (Invitrogen, USA); 2) monitoring DNA degradation and contamination using 1% agarose gels. The Agilent SureSelect Clinical Research Exome Version 2 Probe set kit was applied to perform exome capture. Then, the Illumina HiSeq X platform was used to sequence the captured DNA as instructed in the manufacturer’s protocol, with a paired-end run of 2 × 151 bp in Nantong ZhongKe Medical Laboratory. HCS (HiSeq Control Software v2.2) and RTA (Real Time Analysis. v1.18) were used to analyze initial alignment and quality control. Illumina package bcl2fastq (v1.8.4) was applied to convert the binary base call (BCL) to a FASTQ format. Related sequencing data in this article have been uploaded to the GenBank Data Libraries with accession number as PRJNA684940.

### Bioinformatics Analysis

Short reads (Raw data) in FASTQ format were obtained from fluorescence images on Hiseq platform by base calling. Fastp was used for quality control by discarding low quality paired reads, including reads that contain adapter contamination or more than 10% of uncertain bases in either one read or over 50% low-quality bases in either one read or less than 20 bases after trimming (18). Mapping results were obtained using Burrows-Wheeler Aligner (BWA) and stored in BAM format (19, 20). PCR duplicates of mapped reads were removed with Sambamba (21).

Germline mutations, including single-nucleotide polymorphisms (SNPs) and small insertions and deletions (InDels) of each exome, were detected using the HaplotypeCaller tool of Genome Analysis Toolkit (GATK) software following the protocol (22). To avoid false positives, these criteria were applied to filter.vcf outputs: SNP: “QD< 2.0 || FS > 60.0 || MQ< 40.0 || MQRankSum< -12.5 || ReadPosRankSum< -8.0”, InDel: “QD< 2.0 || FS > 200.0 || ReadPosRankSum< -20.0”. Annotation of filtered SNPs was conducted using ANNOVAR (23) and snpEFF (24). Only mutations that met the following criteria were considered for further analysis (1): the mutation locates in the exon or its flank region ( ± 2bp) (2); its allele frequency is less than 0.01 in GnomAD, 1,000 genomes and in house database (25, 26) (3); it is not a synonymous mutation (4); more than two softwares of SIFT, Polyphen2HDIV, Polyphen2HVAR, MutationTaster2, predict it to be harmful (27–30) (5). Its host gene is predicted to be associated with PC by phenolyzer software (31).

Mutect2 was used to detect somatic mutations, including single-nucleotide variants (SNVs), small insertions and deletions (InDels) (32). Filter Mutect Calls, a tool of GATK, was employed to assess the qualities of all potential variants and only the site with tag “PASS” in the “FILTER” filed were remained. With Vcf2maf (https://github.com/mskcc/vcf2maf) and VEP, vcf files were converted maf files and annotated somatic mutations (33). Cnvkit was used to identify the copy number variants (CNVs) (34).

The relative weights of mutational signatures of in each sample were calculated according to all somatic SNVs. The R package “deconstructSigs,” built on the basis of mutational signatures in COSMIC (http://cancer.sanger.ac.uk/cosmic/signatures), was applied to quantify the contribution of each signature for each tumor (35). The microsatellite instability (MSI) score was calculated withmsisensor2 (36).

The ABSOLUTE algorithm (37) was employed to assess the tumor purity and ploidy of all samples with the default parameters, which took segmented copy-number and MAF profiles as input. The dNdScv R package (38), which is a group of maximum-likelihood dN/dS methods, was used to quantify selection in cancer and somatic evolution in this study.

### Cell Culture and Transfection

BxPC-3 cells were cultured in 1640 medium supplemented with 10% fetal bovine serum (FBS) (Gibco, Thermo Fisher Scientific, Inc, Waltham, MA, USA) and maintained in a humidified 5% CO2 atmosphere at 37˚C. WT (wild type) or Mut (a specific mutation) plasmids of SMAD3 and SMAD4 were purchased from Genechem (Shanghai, China). Briefly, the mutation plasmids were constructed as the mutation information found in the sample (exon2: c.220C>T for SMAD3 gene and exon9: c.1138A>T for SMAD4 gene) by chemical synthesis. To reduce the interference of endogenous expression of SMAD3 or SMAD4, lentiviruses containing SMAD3 or SMAD4 shRNA, purchased from Genechem, were first utilized to knockdown their expression before WT and Mut plasmids transfection in BxPC-3 cells. The siRNAs targeting P21 or FOXO3a were also purchased from Genechem (Shanghai, China).

### Cell Viability Assay and LDH Release Assay

BxPC-3 cells transfected with WT or Mut plasmid were seeded in 96-well plates. We then utilized Cell Counting Kit-8 (CCK-8) from MedChemExpress (Madison, WI, USA) and LDH assay kit (Promega, Madison, WI) to examine cell viability and cytotoxicity detection as the manufacturer’s instructions, respectively. The results were normalized against the Mock group from three independent experiments done in triplicate.

### Colony Formation and Clonogenic Survival

Cell colony formation detection was performed in a 6-well plate. Cells in different groups were seeded at optimal density. Fresh medium was changed every three days. After two weeks, we fixed the cells with methanol and then stained them with crystal violet. The pictures were photographed by a digital scanner. The surviving fraction (SF) was represented by calculating a ratio of the number of colonies formed and the product of the number of cells plated and the plating efficiency.

### Western Blot and Real-Time PCR

Protein extraction, protein determination, Western blot, and real-time PCR were performed as described previously (39).

### Statistical Analyses

Correlation between mutations and clinicohistological variables like overall survival time was measured by statistical R/Bioconductor packages. The significance of differences in data between the groups was determined by the Student’s *t*-test and Fisher’s exact test. With the clusterProfiler package, we performed KEGG pathway enrichment analyses for the significantly genes (40). Statistical significance was set as a *P* value of 0.05 or less.

## Results

### Samples and Clinicopathological Data

Six cases of pancreatic ductal adenocarcinoma (PDAC) patients were involved in this study whose TNM stage ranges from T2 to T4. In our cohort, the mean age of PDAC patients was 66 years (range, 57–77 years; SD, 7.0) and four patients were male (66.7%). With a median follow-up of 17.3 months (SD 24.8), five patients had a dead status (83.3%). According to the response to radiotherapy with a dose of 1.8 to 2, three patients, with the overall survival was beyond 23 months, were considered as a good prognosis, whereas the other three patients were considered as a poor prognosis and died in 11 months (detail in **Table 1**).

### Sequencing Data Summary

Tumor tissues and paired blood samples of six individuals were collected, respectively to conduct the whole exome sequencing. After removing the low-quality, too short and adaptor containing reads, about 48.5 G bases per library were obtained, and the sequence depth ranged from 330X to 533X. Otherwise, the bases and depth of paired samples were 15.4 G and 149X, averagely. Then, all reads were mapped to the UCSC *Homo sapiens* genome (version 19), with the total mapping rate more than 99.5% in all samples. Clean data have the Q30 value more than 90% in our research and fits the need of following identification of somatic mutations (details in **Supplemental Material S1**).

### Somatic Mutations in PDAC

**Figure 1:**
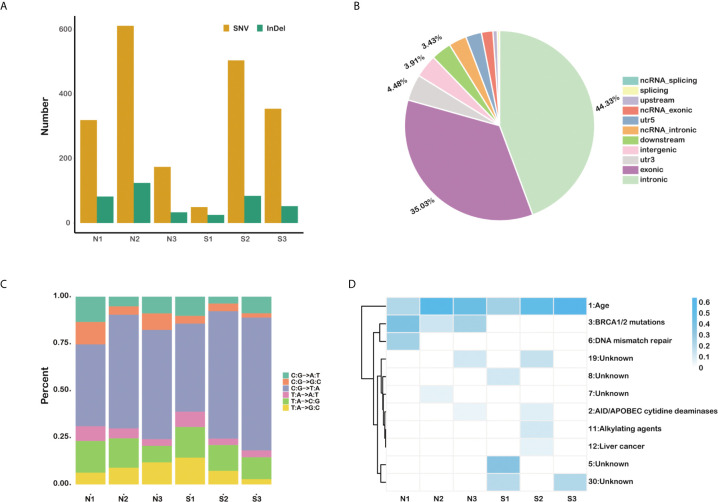
The somatic mutation profiles of six primary pancreatic tumor samples. **(A)** Total number of SNVs and InDels detected in each sample. **(B)** The statistics of somatic mutations in the genome. **(C)** The distribution of mutation type for each sample. **(D)** The mutation signature distribution based on COSMIC signature database.

A total of 2,380 somatic variations (median, 403.5; range, 74–735; SD, 241.3) were identified in the six pancreatic carcinomas samples, including 1,989 single nucleotide polymorphisms and 391 small insertions and deletions (**Figure 1A**). As expected, most variations are located in the exonic region (35.03%) and intronic area (44.33%) of the genomes (**Figure 1B**). We also found that cytosine (C) to thymine (T) transitions were the predominant nucleotide changes (**Figure 1C**). The somatic mutation profiles were analyzed to address the possible mechanism in the occurring and progressing of PDAC (**Figure 1D**). Signature 1, age, has the high weight in all six individuals, and signature 3 enriched in insensitive group members (COSMIC Signatures version 2).

### The TMB, MSI, and Clonality Are Not Related to Radiosensitivity in PDAC

It is known to all that tumor mutational burden and microsatellite instability were closely related with the response to immunotherapy in various types of cancers. To determine whether there are also correlations between tumor mutational burden and radiosensitivity in PDAC patients, we examined the number of all mutations in the target region as tumor mutation burden in this tumor type. As shown in **Table 2**, our results showed that there was no significant correlation between overall survival time (OS) and MSI (p=0.9194). Otherwise, the TMB was negatively correlated with the OS with a coefficient of -0.66 (p=0.175). To determine whether radiosensitivity was correlated with clonality, the purity and copy number alterations were then analyzed in this study. Our results indicated that there was no obvious association between OS and purity or ploidy (r = 0.029424 and r = 0.2, respectively) (**Table 2**). These results showed that the overall survival is not significantly correlated with the number of missense mutations, the instability of microsatellite, and clonality in PDAC patients treated with radiotherapy. The possibly influencing factors, age and tumor stage, were also analyzed (**Table 2**). However, the results showed that all these factors are not the core factors causing radioresistance in PDAC.

**Table 2:** Difference of TMB, MSI, and clonality between sensitive and insensitive groups.

Type	N1	N2	N3	S1	S2	S3	Correlation	p value
TMB	5.78	10.37	2.96	1.04	8.29	5.75	−0.6571429	0.175
MSI (%)	5.95	6.38	5.33	5.53	5.36	7.41	0.08571429	0.9194
Purity	0.21	0.2	0.2	0.18	0.21	0.24	0.029424	0.9559
Ploidy	2.27	1.86	3.56	2.04	3.98	2.98	0.2	0.7139
Age	71	63	65	77	57	63	0.02898855	0.9565
Stage	3	1	2	2	3	2	−0.277746	0.5941

### Differences Between Sensitive and Insensitive Individuals in PDAC

**Figure 2:**
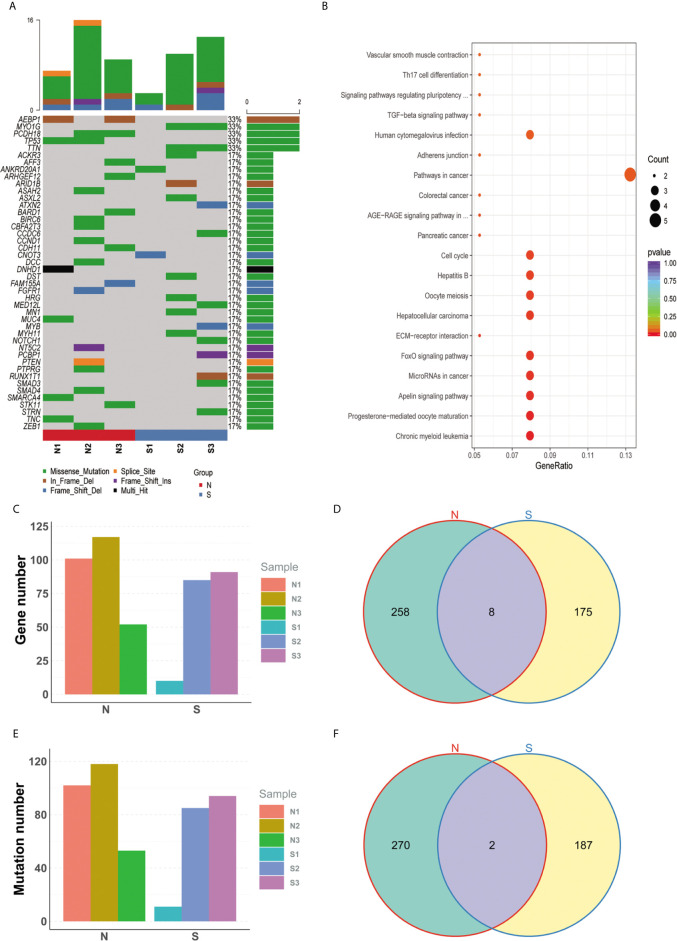
Specific mutated genes between group N and group S. **(A)** The oncoprint plot of all somatic mutations. **(B)** The bubble chart of kegg terms enriched for all diff-mutated genes. **(C)** No significant difference on total number of genes was observed between group N group and group S. **(D)** There are 8 common genes in both groups, as well as 258 and 175 unique genes belonged to the group N and group S, respectively. **(E)** No significant difference on total number of variations was observed between group N and group S. **(F)** 2 common mutations were shared in both groups, but 270 and 187 unique mutations belonged to the group N and group S, respectively.

Somatic mutations were filtered according to the criterions mentioned in the method to expose the candidate deleterious sites. 459 in 2301 variants remained, which is located in 441 genes, such as TP53, KRAS, PTEN, and so on (**Figure 2A**, detail in **Supplemental Material S2**). We next counted the number of variants for each gene in sensitive and insensitive members. Among them, 434 genes were only mutated in group N or group S, which were defined to be radiosensitivity-related genes. To address the possibly functions that these radiosensitivity-related genes took part in, Gene Ontology and KEGG enrichment analysis were conducted (**Figure 2B**). The most significant GO terms contained response to extracellular stimulus, mitotic cell cycle checkpoint, DNA catabolic process, and so on (detail in **Supplemental Material S3**). Pathway terms, like cell cycle, TGF-beta signaling pathway, and FoxO signaling pathway, were significantly enriched in our study, which might be closely related with radiosensitivity in PDAC (detail in **Supplemental Material S4**). As shown in **Figures 2C, E**, no significant differences on total number of genes and variation were observed between group N and group S. There are eight common genes in both groups, as well as 258 and 175 unique genes belonged to the group N and group S, respectively (**Figure 2D**). Moreover, two common mutations were shared in both groups, but 270 and 187 unique mutations belonged to the group N and group S, respectively (**Figure 2F**). Germline mutations were also analyzed in our study. All the mutations were filtered by the procedures mentioned in the method, and 754 sites in 639 genes remained (Details in **Supplemental Material S5**).

### Pathway-Based Protein Interaction Network of Radiosensitivity-Related Genes

**Figure 3:**
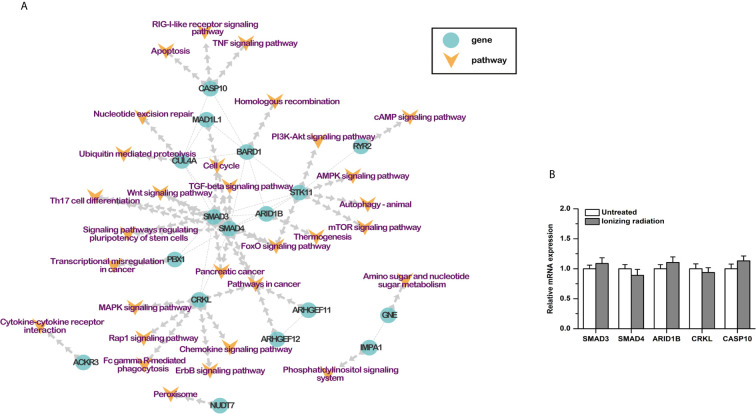
**(A)** Protein-protein interaction network of RI-related genes. **(B)** The mRNA expression of SMAD3, SMAD4, ARID1B, CRKL, and CASP10 was not affected by ionizing radiation (4Gy) in PDAC cells.

Based on previous studies, 30 RI-related pathways were selected, including cell cycle, homologous recombination, nucleotide excision repair, and so on. Combined with the protein-protein interactions, a pathway-based protein-protein interaction (PPI) network was constructed with the RI-related genes (**Table 3**). Seventeen genes were involved. SMAD3, SMAD4, ARID1B, CRKL, and CASP10 played a center role in the network (**Figure 3A**). Moreover, the results of qPCR showed that the mRNA expression of these five genes was not affected by ionizing radiation in BxPC-3 cells (**Figure 3B**). It is worth noting that both SMAD3 and SMAD4 belong to the SMAD proteins family, which is a group of intracellular signal transducer proteins. The synonymous mutations of somatic mutations are listed in **Supplemental Material S6**.

**Table 3:** RI Related Pathways of Somatic Mutation Related Genes.

ID	Description	Gene Ratio	BgRatio	pvalue	p.adjust	geneName	kegg ID	Count
hsa04068	FoxO signaling pathway	3/38	73/5345	0.01464519	0.262881855	SMAD3/SMAD4/STK11	hsa:4088/hsa:4089/hsa:6794	3
hsa04110	Cell cycle	3/38	90/5345	0.025474288	0.262881855	MAD1L1/SMAD3/SMAD4	hsa:8379/hsa:4088/hsa:4089	3
hsa05212	Pancreatic cancer	2/38	38/5345	0.029462868	0.262881855	SMAD3/SMAD4	hsa:4088/hsa:4089	2
hsa05200	Pathways in cancer	5/38	280/5345	0.046367277	0.262881855	ARHGEF11/ARHGEF12/CRKL/SMAD3/SMAD4	hsa:9826/hsa:23365/hsa:1399/hsa:4088/hsa:4089	5
hsa04350	TGF-beta signaling pathway	2/38	50/5345	0.048688233	0.262881855	SMAD3/SMAD4	hsa:4088/hsa:4089	2
hsa04550	Signaling pathways regulating pluripotency of stem cells	2/38	51/5345	0.050452728	0.262881855	SMAD3/SMAD4	hsa:4088/hsa:4089	2
hsa04659	Th17 cell differentiation	2/38	51/5345	0.050452728	0.262881855	SMAD3/SMAD4	hsa:4088/hsa:4089	2
hsa04310	Wnt signaling pathway	2/38	77/5345	0.103329175	0.385293447	SMAD3/SMAD4	hsa:4088/hsa:4089	2
hsa00520	Amino sugar and nucleotide sugar metabolism	1/38	16/5345	0.1080262	0.385293447	GNE	hsa:10020	1
hsa03440	Homologous recombination	1/38	24/5345	0.157689869	0.437818785	BARD1	hsa:580	1
hsa03420	Nucleotide excision repair	1/38	27/5345	0.175610754	0.437818785	CUL4A	hsa:8451	1
hsa04622	RIG-I-like receptor signaling pathway	1/38	32/5345	0.204657144	0.45599297	CASP10	hsa:843	1
hsa04012	ErbB signaling pathway	1/38	40/5345	0.249070673	0.466593073	CRKL	hsa:1399	1
hsa04070	Phosphatidylinositol signaling system	1/38	43/5345	0.2650952	0.466593073	IMPA1	hsa:3612	1
hsa04146	Peroxisome	1/38	44/5345	0.270362342	0.466593073	NUDT7	hsa:283927	1
hsa04152	AMPK signaling pathway	1/38	44/5345	0.270362342	0.466593073	STK11	hsa:6794	1
hsa04668	TNF signaling pathway	1/38	54/5345	0.321052415	0.484828318	CASP10	hsa:843	1
hsa04666	Fc gamma R-mediated phagocytosis	1/38	63/5345	0.363729772	0.511477378	CRKL	hsa:1399	1
hsa04210	Apoptosis	1/38	79/5345	0.433240846	0.558515308	CASP10	hsa:843	1
hsa04024	cAMP signaling pathway	1/38	83/5345	0.449428355	0.564119374	RYR2	hsa:6262	1
hsa04150	mTOR signaling pathway	1/38	83/5345	0.449428355	0.564119374	STK11	hsa:6794	1
hsa04062	Chemokine signaling pathway	1/38	87/5345	0.46516535	0.565598778	CRKL	hsa:1399	1
hsa05202	Transcriptional misregulation in cancer	1/38	99/5345	0.509793746	0.590622294	PBX1	hsa:5087	1
hsa04015	Rap1 signaling pathway	1/38	100/5345	0.513344611	0.590622294	CRKL	hsa:1399	1
hsa04120	Ubiquitin mediated proteolysis	1/38	104/5345	0.527299295	0.600223665	CUL4A	hsa:8451	1
hsa04060	Cytokine-cytokine receptor interaction	1/38	117/5345	0.570013689	0.628777987	ACKR3	hsa:57007	1
hsa04010	MAPK signaling pathway	1/38	141/5345	0.639214606	0.697917988	CRKL	hsa:1399	1
hsa04151	PI3K-Akt signaling pathway	1/38	189/5345	0.746619194	0.790972809	STK11	hsa:6794	1
hsa04140	Autophagy—animal	1/38	212/5345	0.786339323	0.818519946	STK11	hsa:6794	1
hsa04714	Thermogenesis	1/38	342/5345	0.91968023	0.928356458	ARID1B	hsa:57492	1

### Mutations of SMAD3 and SMAD4 Affect the Radiosensitivity in PDAC

**Figure 4:**
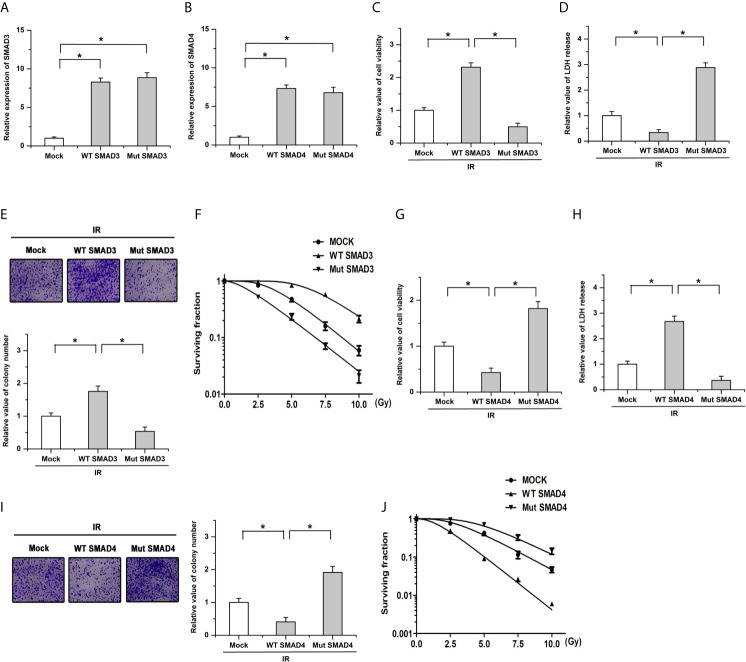
The radiosensitivity is affected by the mutation of SMAD3 or SMAD4 in PDAC. **(A, B)** Results of qPCR showed that there were no obvious differences on the expression of SMAD3 **(A)** or SMAD4 **(B)** between the WT and Mut cells, respectively. **(C, D)** The regulatory effects of IR on cell viability **(C)** and LDH release **(D)** were facilitated by the SMAD3 mutation. **(E, F)** The decreases of colony formation **(E)** and surviving fraction **(F)** induced by IR were enhanced by the mutation of SMAD3 compared with the WT group. **(G, H)** SMAD4 mutation mitigated the effects of IR on cell survival **(G)** and LDH release **(H)**. **(I, J)** The decrease of colony formation **(I)** and surviving fraction **(J)** induced by IR were antagonized by SMAD4 mutation compared with the WT group. Cells infected with the empty vector were defined as the mock group. *P < 0.05.

The ectopic wild-type (WT; without the variation) and mutant (Mut; with the variation) SMAD3 or SMAD4 cells were constructed in BxPC-3 cells (a PDAC cell line) to determine whether the mutation of SMAD3 or SMAD4 affected the radiosensitivity. As shown in **Figures 4A, B**, results of qPCR showed that there were no obvious differences on the expression of SMAD3 or SMAD4 between the WT and Mut cells, respectively. We then examined cell survival and death after ionizing radiation (IR) by utilizing CCK-8 and LDH release assay. The results showed that transfection of WT SMAD3 repressed the decreases of cell viability and the increase of LDH release induced by ionizing radiation (IR, 4Gy), whereas mutation of SMAD3 facilitated the effects of IR on cell survival and death (**Figures 4C, D**). Moreover, transfection of SMAD3 mutation significantly promoted IR-induced decreases of colony formation and surviving fraction in PDAC cells compared with the WT group (**Figures 4E, F** **)**. On the contrary, the effects of IR on cell survival, cell death, colony formation, and surviving fraction were inhibited by the mutation of SMAD4 compared with the WT group (**Figures 4G–J**). These results indicate that SMAD3 mutation or SMAD4 mutation leads to the different response to radiotherapy in PDAC.

### Effects of SMAD3 and SMAD4 on Radiosensitivity Are Mediated by Different Signaling Pathways

**Figure 5:**
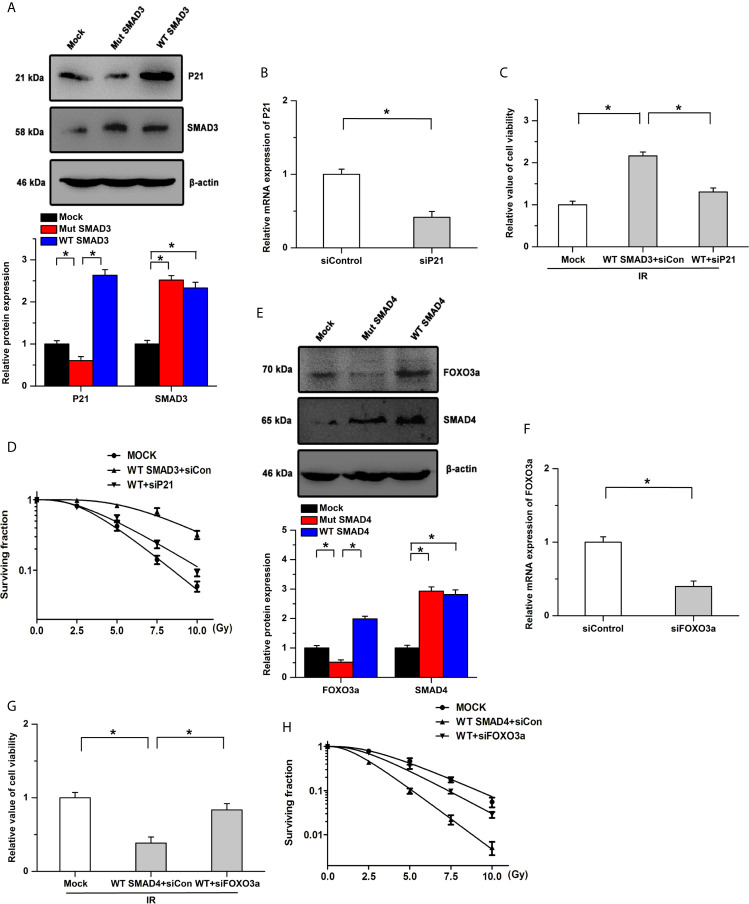
Effects of SMAD3 and SMAD4 on radiosensitivity are mediated by different signaling pathways. **(A)** Expression of P21 was significantly increased by transfection of WT SMAD3, while the effects were abolished by the mutation of SMAD3. **(B)** Knockdown efficiency of siP21 was verified by Real-time PCR. **(C, D)** SMAD3 inhibits IR-induced the decreases of cell viability **(C)** and surviving fraction **(D)**, which were mitigated by P21 knockdown. WT indicates WT SMAD3, siCon indicates siControl. **(E)** Transfection of WT SMAD4, but not SMAD4 mutation, increased the expression of FOXO3a. **(F)** Real-time PCR was utilized to determine the knockdown efficiency. **(G, H)** Inhibitory effects of SMAD4 on the decreases of cell viability **(G)** and surviving fraction **(H)** induced by IR were attenuated by the knockdown of FOXO3a. WT indicates WT SMAD4, siCon indicates siControl. *P < 0.05.

Previous studies have indicated that SMAD3 directly binds to the promoter of cyclin-dependent kinase inhibitor 1 (P21) and SMAD4 could regulate the activity and expression of FOXO3a by forming a complex (41, 42). Based on the protein-protein interaction (PPI) network, SMAD3 and SMAD4 could regulate cell cycle and the FOXO signaling pathway, respectively. We then determined whether SMAD3 and SMAD4 regulated the radiosensitivity by P21 and FOXO3a in PDAC. Our results showed that SMAD3 overexpression facilitates the expression of P21, whereas the effects were abolished by the mutation of SMAD3 (**Figure 5A**). The inhibitory effects of SMAD3 on the decrease of cell viability and surviving fraction were attenuated by the knockdown of P21 (**Figures 5B–D**). On the other hand, the expression of FOXO3a was increased by transfection of WT SMAD4, whereas mutation of SMAD4 had no detectable effects on its expression (**Figure 5E**). Overexpression of SMAD4 mitigated cell viability and surviving fraction after IR treatment, which was attenuated by the knockdown of FOXO3a (**Figures 5F–H**). These results indicate that SMAD3 and SMAD4 regulate the radiosensitivity of PDAC, at least in part, by P21 and FOXO3a, respectively.

## Discussion

Whole-exosome mutations profiles of PDAC were analyzed in this study. Somatic mutations locate in 441 genes were detected to be radiosensitivity-related loci. Functional enrichment and pathway-based protein-protein interaction analysis were used to address the possible mechanism involved in RI, 17 genes and 15 related pathways were identified to likely influence the radiosensitivity in PDAC. Furthermore, effects of IR on cell growth and colony formation were facilitated by the mutation of SMAD3, whereas SMAD4 mutation had the opposite effects in PDAC. The results indicate that SMAD3 and SMAD4 likely cause the difference of response to radiotherapy in PDAC.

Accumulating evidence has indicated that many mutational signatures exists in various types of tumors. It has been revealed that the unique combinations of mutation types resulted from different mutational processes (43). Consistent with most other cancer studies, all members enriched in the signature of age (signature 1), which is the result of an endogenous mutational process initiated by spontaneous deamination of 5-methylcytosine (44). Interestingly, all three insensitive group members exhibited an enrichment of the signature 3 (COSMIC Signatures version 2), which has been approved to be related with the failure of DNA double-strand break-repair by homologous recombination (45). However, germline and somatic BRCA1 and BRCA2 mutations, the proven failure causes of DNA double-strand break-repair, were not detected in our cohort, further studies would be done to explore the implicit mechanism.

As is known, the TMB and MSI score are reliable biomarkers of immunotherapy (46, 47). We analyzed the correlation between TMB, MSI, and OS and found that they were not obviously correlated, suggesting that the radiosensitivity might not be decided by these biomarkers. In the study of drug sensitivity, no significant association is found between OS and mutation burden or clonality in gastric cancer (48), whether it is the same in RI is still not studied. In this study, clonality, age, and tumor stage were also not the reason of RI in PDAC. All these results confirm that the mechanism in RI might be different with the other therapies.

The Smad protein family acts as the crucial mediators in the TGF-β signaling pathway, could transmit signals from the cell surface to the nucleus, and regulates gene activity and cell proliferation. As a member of Smad family, SMAD3 plays an important role in cancer progression, and its mutations status and DNA methylation level were related to the development of various kinds of cancers (49). Ken et al. found that increased expression of SMAD3 facilitates epithelial-mesenchymal transition and leads to poor prognosis in PDAC (50). In our study, SMAD3 was mutated in the radiosensitive group. Mutation of SMAD3 promoted the effects of IR on cell growth and colony formation in PDAC cells. SMAD4 also belongs to the Smad family same family and could form heteromeric complexes with other activated Smad proteins. The heteromeric complexes translocate and accumulate in the nucleus and then modulate the transcription of the target genes. It is reported that SMAD4 inhibits epithelial cell proliferation by acting as a tumor suppressor (51). The occurrences of pancreatic cancer, juvenile polyposis syndrome, and hereditary hemorrhagic telangiectasia syndrome are closed related with the mutations or deletions of SMAD4 (52–54). Previous studies have shown that resistance to radiotherapy likely resulted from the absence or low expression level of TGF-beta receptor or SMAD4 (15, 55). Wang F et al. have found that defective SMAD4 lead to the radioresistance by increasing persistently higher levels of ROS and promoting the autophagy caused by radiation in pancreatic cancer (55). In the present study, mutation of SMAD4 was found in the radioresistance group of PDAC, and SMAD4 acted as the central molecule in radioresistance. Mutation of SMAD4 mitigated the effects of IR on cell growth in PDAC, which is consistent with a previous study. However, further studies are still needed to elucidate the mechanism of SMAD3/SMAD4 on the response to radiotherapy in PDAC.

Furthermore, our results also showed that some PPI associated to SMAD genes might be associated with the radiosensitivity in PDAC. ARID1B, which encodes an AT-rich DNA interacting domain-containing protein, is a component of the SWI/SNF chromatin remodeling complex and may play a role in cell-cycle activation and influence DNA damage response. Yang L et al. found that depletion of ARID1A significantly enhances the radioresistance of pancreatic cancer cells and activates the PI3K/AKT signaling pathway (56). It is reported that depletion of ARID1B sensitizes colorectal cancer cells with ARID1A mutation to ionizing radiation (57). Inhibition of either ARID1A or ARID1B increases cellular sensitivity to ionizing radiation, and SWI/SNF factors are required for cellular resistance to ionizing radiation (58). CUL4A, belonging to the cullin family subunit of ubiquitin-protein ligases, inhibits the cellular repair capacity and the cell cycle checkpoint in response to DNA damage. Previous studies have shown that CUL4A overexpression renders MCF10A more sensitive to ionizing radiation, and knockdown of CUL4A facilitates the global genomic repair pathway and augments DNA damage response induced by ultraviolet (UV) irradiation (59, 60). Cellular sensitivity to ionizing radiation is decreased by CUL4A depletion by inhibiting the degradation of Chk1 (61). STK11 often serves as a tumor suppressor in some cancers. However, the potential functions of STK11 in response to IR are not always consistent. It is reported that STK11-activated autophagy enhances resistance to the combination of trametinib and radiation in KRAS-mutant NSCLC and promotes esophageal squamous cell carcinoma cell survival after radiation (62, 63). Conversely, inhibition of the STK11-SIK1 signaling pathway promotes epithelial-mesenchymal transition and radioresistance in NSCLC (64). In addition, CRKL, frequently unregulated in several malignant tumors, positively regulates the progression of cancers by promoting cell proliferation and metastasis (65). Knockdown of PBX1 significantly enhanced the radiosensitivity *via* STAT3 in esophageal squamous cancer (66). In our results, we found that mutations of CUL4A, STK11, and CRKL were acquired in the radioresistance group in PDAC, whereas mutation of ARID1B and PBX1 were found in the radiosensitive group. However, the concrete roles and corresponding mechanism of these genes in regulating the radiosensitivity of PDAC still need to be determined in further studies.

Meanwhile, this study had some limitations. Although we have identified that some key genes mutations might be associated with the radiosensitivity and proved that SMAD3 and SMAD4 affected radiosensitivity through different signaling pathways in PDAC *in vitro*, the number of patients used in our study is small, which may cause data bias in sequencing results. Further studies are needed to validate the effects of these key mutations on radiosensitivity of pancreatic cancer in more clinical samples and *in vivo* study. Moreover, it is necessary to determine whether the radiosensitivity is also affected by the mutations of SMAD3 or SMAD4 in other cancers, such as nasopharyngeal carcinoma and lung cancer.

## Conclusions

In summary, our results have identified several genes mutations associated with the radiosensitivity in PDAC. Moreover, SMAD3 and SMAD4 genes mutate differently between radiosensitive and radioresistance patients with PDAC, implying that SMAD3 and SMAD4 participate in regulating the radiosensitivity in PDAC by different pathways. These results might provide some biomarkers and potential targets for the radiotherapy of PDAC patients in the future.

## Data Availability Statement

The datasets presented in this study can be found in online repositories. The names of the repository/repositories and accession number(s) can be found below: https://www.ncbi.nlm.nih.gov/genbank/, PRJNA684940.

## Ethics Statement

The studies involving human participants were reviewed and approved by Renji Hospital. The patients/participants provided their written informed consent to participate in this study.

## Author Contributions

YB, JM and LZ designed this study. BH, RH, ZW, CM, YG and JL analyzed the data. LZ and CM wrote the manuscript. JT and XM collected the data. JM, XM and LZ revised the manuscript. All authors contributed to the article and approved the submitted version.

## Funding

This study was sponsored by National Natural Science Foundation of China (grant no. 81802626 and 81972854), the Incubating Program for Clinical Research and Innovation of Renji Hospital (grant no. PYII-17-006), Science and Technology Commission of Shanghai Municipality (grant no. 21ZR1438500).

## Conflict of Interest

The authors declare that the research was conducted in the absence of any commercial or financial relationships that could be construed as a potential conflict of interest.

## Publisher’s Note

All claims expressed in this article are solely those of the authors and do not necessarily represent those of their affiliated organizations, or those of the publisher, the editors and the reviewers. Any product that may be evaluated in this article, or claim that may be made by its manufacturer, is not guaranteed or endorsed by the publisher.
